# Genome-wide detection of predicted non-coding RNAs in *Rhizobium etli *expressed during free-living and host-associated growth using a high-resolution tiling array

**DOI:** 10.1186/1471-2164-11-53

**Published:** 2010-01-20

**Authors:** Maarten Vercruysse, Maarten Fauvart, Lore Cloots, Kristof Engelen, Inge M Thijs, Kathleen Marchal, Jan Michiels

**Affiliations:** 1 Centre of Microbial and Plant Genetics, Katholieke Universiteit Leuven, B-3001 Heverlee, Belgium

## Abstract

**Background:**

Non-coding RNAs (ncRNAs) play a crucial role in the intricate regulation of bacterial gene expression, allowing bacteria to quickly adapt to changing environments. In the past few years, a growing number of regulatory RNA elements have been predicted by computational methods, mostly in well-studied γ-proteobacteria but lately in several α-proteobacteria as well. Here, we have compared an extensive compilation of these non-coding RNA predictions to intergenic expression data of a whole-genome high-resolution tiling array in the soil-dwelling α-proteobacterium *Rhizobium etli*.

**Results:**

Expression of 89 candidate ncRNAs was detected, both on the chromosome and on the six megaplasmids encompassing the *R. etli *genome. Of these, 11 correspond to functionally well characterized ncRNAs, 12 were previously identified in other α-proteobacteria but are as yet uncharacterized and 66 were computationally predicted earlier but had not been experimentally identified and were therefore classified as novel ncRNAs. The latter comprise 17 putative sRNAs and 49 putative *cis*-regulatory ncRNAs. A selection of these candidate ncRNAs was validated by RT-qPCR, Northern blotting and 5' RACE, confirming the existence of 4 ncRNAs. Interestingly, individual transcript levels of numerous ncRNAs varied during free-living growth and during interaction with the eukaryotic host plant, pointing to possible ncRNA-dependent regulation of these specialized processes.

**Conclusions:**

Our data support the practical value of previous ncRNA prediction algorithms and significantly expand the list of candidate ncRNAs encoded in the intergenic regions of *R. etli *and, by extension, of α-proteobacteria. Moreover, we show high-resolution tiling arrays to be suitable tools for studying intergenic ncRNA transcription profiles across the genome. The differential expression levels of some of these ncRNAs may indicate a role in adaptation to changing environmental conditions.

## Background

The first bacterial non-coding RNAs (ncRNAs) were discovered over 25 years ago [1,2]. Still, only in the past decade have we begun appreciating their crucial role in bacterial gene regulation in response to environmental changes. By controlling metabolic pathways or stress responses, these ncRNAs play a role in diverse biological processes, including regulation of outer membrane proteins or transporters, iron metabolism, pathogenesis, quorum sensing and plasmid copy number [3-7].

The regulatory mode of action of ncRNAs is diverse as well. The best characterized group of RNA regulators are short transcripts termed small RNAs (sRNAs) that regulate gene expression through base pairing with mRNA and are either *cis*- or *trans*-encoded [8]. Other ncRNAs can bind to proteins in order to modulate protein activity [9]. Regulatory RNAs also include mRNA leader sequences that control expression of the downstream genes. These *cis*-regulatory RNA elements can be antisense RNA controlling mRNA transcription or 5' untranslated regions (UTRs) modulating expression through conformation changes by temperature shift or binding of specific metabolites [10,11]. This kind of regulation can lead to premature transcription termination of the 5' UTR, concomitantly producing a short transcript. Recently, a new group of RNA regulators was discovered, called CRISPR (clustered regulatory interspaced short palindromic repeats) RNAs. These RNAs provide resistance to bacteriophage infection and prevent plasmid conjugation [12].

Although just a small number of ncRNAs were known in *E. coli *initially, the use of computational predictions changed this dramatically [13-16]. Today, over 80 sRNAs are known in *E. coli*. In recent years the search was extended to many more bacterial species such as *Bacillus subtilis*, *Vibrio cholerae*, *Pseudomonas aeruginosa*, *Staphylococcus aureus*, *Streptomyces coelicolor*, *Salmonella enterica*, *Mycobacterium tuberculosis *and *Listeria monocytogenes *[17-23]. The experimental approaches for identification include computational predictions, direct detection by dedicated microarrays or Northern blotting, direct isolation (RNomics), co-purification with RNA-binding proteins and high-throughput pyrosequencing [24,25]. In addition, advances in array technology and the growing list of sequenced microbial replicons make custom-design high-density arrays increasingly affordable and attractive for a multitude of organisms, and expression-based ncRNA discovery and transcription profiling on a genome-wide scale feasible. Still, this was not put into practice until very recently [26,27].

In this study, we used a high-resolution tiling array representing the entire genome of *Rhizobium etli*, the nitrogen-fixing endosymbiont of the common bean plant *Phaseolus vulgaris *[28,29], to perform a focused study of transcriptionally active intergenic regions (IGR). Loci showing significant expression were compared to an extensive compilation of recently published ncRNA predictions in *R. etli *and related α-proteobacteria. 89 candidate ncRNAs similar to one or more predicted or previously detected ncRNAs were detected, and a selection of these was confirmed by Northern analysis and 5' RACE. Numerous ncRNAs are differentially expressed in *R. etli *during free-living growth and symbiosis with the eukaryotic host. Our results therefore significantly expand the known repertoire of ncRNAs in α-proteobacteria and provide a wealth of information for future studies to build on.

## Methods

### Bacterial strains and growth conditions

**Figure 1:**
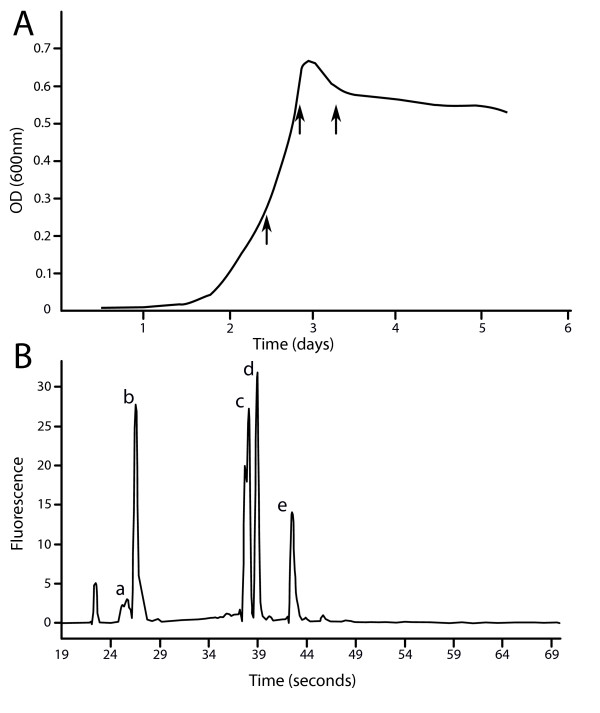
**Sampling conditions and rRNA fragmentation**. (A) Three samples were taken during free-living growth based on the OD of the culture; early exponential phase (OD_600 _= 0.3), late exponential phase (OD_600 _= 0.7) and stationary phase (6 hours after reaching the maximum OD_600_). (B) An example of high quality total RNA, illustrating the fragmentation 23S rRNA: (a) small RNA peak including 5S rRNA and the ncRNAs (b) 23S fragment of ~135 bp (c) two 23S fragments of ~1300 bp (d) 16S rRNA (e) intact 23S rRNA.

In order to study expression in the free-living state, wild-type *R. etli *CFN42 was grown at 30°C in acid minimal salts medium supplied with 10 mM NH_4_Cl and 10 mM succinate while monitoring the optical density (OD) of the culture [30]. Samples were taken at OD_600 _= 0.3, 0.7 and 6 hours after reaching the maximum OD, representing early/late exponential and stationary phase, respectively (Figure 1A). In order to study gene expression during host-associated growth, common bean plants (*Phaseolus vulgaris *cv. Limburgse vroege) were cultivated and inoculated as described previously [31,32]. Nodules were harvested 2 and 3 weeks after inoculation and the bacteroids were purified by differential centrifugation.

### RNA isolation and detection by tiling microarray

Total RNA was isolated without sRNA enrichment by adapting previously published protocols [33-35]. The RNA content of 20 ml and 40 ml bacterial culture in respectively exponential and stationary phase was stabilized by adding 1/5 volume of ice-cold phenol:ethanol (5:95). Cells were harvested by centrifugation, pellets were immediately frozen in liquid nitrogen and stored at -80°C. Liquid nitrogen was used to flash freeze nodules and to crush them with a mortar and pestle. Each sample of 10 g powder, obtained from 25 plants, was suspended in 1 volume of RNAprotect (Qiagen) and 2 volumes of 10 mM MgSO_4 _to further stabilize the bacteroid RNA. Bacteroid and plant material was separated by differential centrifugation. The bacteroid pellet was immediately frozen in liquid nitrogen and stored at -80°C.

Total RNA was extracted using the TRIzol Plus RNA Purification kit (Invitrogen). The cell or bacteroid pellets were resuspended in 1 ml of TRIzol and shaken twice by a Precellys 24 (Bertin Technologies) at 6500 rpm for 45 seconds with 0.25 ml of 0.1 mm glass beads before following the manufacturer's instructions. Phase Lock Gel tubes (heavy type) were used to efficiently separate the organic and aqueous phase. DNA contamination was removed by two treatments of 2 μl TURBO DNase (Ambion) and afterwards checked by PCR (45 cycles). To increase RNA yields and account for experimental variation, RNA from 6 different cultures or 4 batches of bacteroids was pooled. RNA was precipitated in 3 volumes of isopropanol and 1/10 volume of sodium acetate, washed twice in ethanol and dissolved in nuclease-free ultrapure water. RNA integrity was analyzed using Experion RNA StdSens Chips (Biorad, Hercules, CA, before and after precipitation) [36]. All samples had an RNA Quality Indicator value of 10. The ncRNA peak could be detected in each sample (Figure 1B). RNA quantity and purity was assessed using the NanoDrop ND-1000. The A260/A280 ratio and A260/A230 ratio of all samples were ≥ 2.

cDNA was synthesized using random decamers (Ambion) and the SuperScript Double-Stranded cDNA Synthesis Kit (Invitrogen) according to the manufacturer's protocol.

A whole-genome tiling array covering the entire *R. etli *genome sequence (6.5 Mbp in total) was designed by NimbleGen Systems, Inc. (Madison, WI), with ~385,000 60mer probes having an average start-to-start spacing of 13 base pairs and consequently an average overlap of 47 base pairs. Samples were hybridized and scanned by NimbleGen Systems [37,38].

### Microarray data preprocessing

Data preprocessing was done by performing a nonlinear intensity-dependent rescaling on non-background corrected data. To this end, a loess fit normalization [39] with a span of 25% was performed for each array compared against an artificial reference array, consisting of the median intensity values of each probe across all arrays. To ensure that the artificial reference itself was not altered by this rescaling, the artificial reference expression levels were chosen for the average log intensity in the loess fit (instead of the mean expression levels of the respective array and the artificial reference). A robust median-polish procedure was used to combine measurements from multiple probes into a single value [40].

The data were deposited in the NCBI Gene Expression Omnibus (GEO) and can be accessed through accession numbers GSE18580, GSM462173, GSM462178, GSM462180, GSM462184 and GSM462187.

### ncRNA detection

A list of 1814 computationally predicted ncRNAs in *R. etli *and other α-proteobacteria was compiled from literature [26,41-45]. This list was used as a query in a BLASTN search against all *R. etli *intergenic regions (IGR) ≥60 bp with an E-value threshold of 10^-5 ^and with otherwise default parameters, resulting in 447 non-redundant candidate ncRNA regions. The IGRs were extracted from the NCBI Genome database and defined as the regions separating annotated genes. To determine the significance of the obtained expression in each condition, a robust estimation of the noise in the expression data was carried out for each experiment (red curve, see Additional file 1, Figure S1). The cut off (red vertical line, see Additional file 1, Figure S1) was based on the normal inverse cumulative distribution function at 99.9%. 89 regions corresponding to putative ncRNAs were found to be expressed above this significance threshold (see Additional file 2, Table S1). Differential expression across the five conditions was determined by applying the same procedure to the distribution of the standard deviations of the normalized intensities over all conditions (see Additional file 3, Figure S2). Every significantly expressed candidate ncRNA was used to query the Rfam database and RibEx (Riboswitch Explorer) web server [46]. RibEx compares query sequences to known riboswitches as well as other predicted, but highly conserved, bacterial regulatory elements. Detailed information on the analysis of the candidate ncRNAs is summarized in Additional file 2, Table S1.

### RT-qPCR

Expression levels were verified by reverse transcription quantitative real-time PCR (RT-qPCR) using the StepOnePlus System and Power SYBR Green PCR Master Mix containing AmpliTaq Gold DNA Polymerase (Applied Biosystems). Primers were designed using Primer Express 3.0 (optimal primer length of 20 bases, GC-content of 40-60% and Tm of 58-60°C, see Additional file 4, Table S2) and purchased from MacroGen. Secondary structures and dimer formation were checked with Oligoanalyzer 3.1. 2 μg of pooled total RNA of each growth condition (early/late exponential phase, stationary phase, symbiotic state 2 and 3 weeks after inoculation) was reverse transcribed to single stranded cDNA using the SuperScript VILO cDNA Synthesis Kit, including SuperScript III Reverse Transcriptase and random primers, according to the manufacturer's instructions (Invitrogen). DNA contamination of the RNA samples was checked by PCR (45 cycles) before RT and a negative control without cDNA template was included during qPCR. cDNA was stored at -80°C and stock solutions were prepared to minimize freeze-thaw cycles. After dilution of cDNA, 2 μl of cDNA (20 ng/μl), 2 μl of each specific primer (200 nM) and 4 μl of nuclease-free water were mixed with 10 μl of Power SYBR Green PCR Master Mix. In order to confirm that there was no background contamination, a negative control was included in each run. PCR conditions were: a holding stage of 10 min at 95°C, a cycling stage of 45 cycles of 15 s at 95°C and 1 min at 60°C and a melting curve stage of 15 s at 95°C, 1 min at 60°C increased to 95°C with steps of 0.3°C. The last stage was used to verify the specificity of each PCR reaction. All reactions were performed in triplicate and carried out in fast optical 96-well reaction plates (MicroAmp using optical adhesive film (MicroAmp)) with heat bonding as the method of sealing. The absence of inhibitors and the efficiency of each primer were determined by standard curves with dilution series of cDNA (5 log_10 _concentrations) in each run for the reference gene and the ncRNA of interest. The calibration curve's linear interval included the interval for each ncRNA of interest being quantified. The raw data was analyzed using StepOne Software v2.1. 16S RNA (RHE_CH00059) showed an invariant expression under the experimental conditions and was used as reference gene. The early exponential phase was used as calibrator condition. Relative gene expression was calculated using the Pfaffl method that corrects for differences in amplification efficiency [47].

### Northern analysis

Northern hybridization was performed using 1 μg of single strand DNA probe 5' end-labeled with digoxigenin (see Additional file 4, Table S2). RiboRuler RNA Ladder, Low Range (Fermentas) was used to estimate the sizes of the RNA bands. Total RNA (10-15 μg) was separated on 6% PAGE-urea gels and transferred to Hybond-N nylon membranes (Amersham) by electroblotting. The membranes were hybridized overnight in ULTRAhyb-Oligo Buffer (Ambion) at 42°C. After hybridization, membranes were first washed with buffer 1 (100 mM maleic acid, 150 mM NaCl, 7 g NaOH, pH 7.5) followed by a wash with buffer 2 (5 ml blocking stock (10% blocking reagent in buffer 1) and 45 ml buffer 1). 4 μl of anti-dioxigenin AP-Fab fragments (Roche) were added to 20 ml of buffer 2 and incubated at room temperature for 30 min. Unbound antibodies were removed by two washes with buffer 1. The membrane was equilibrated for 2 min with buffer 3 (100 mM Tris-HCl, 100 mM NaCl, 50 mM MgCl_2_, pH 9.5). Finally, 10 μl of chloro-5-substituted adamantyl-1,2-dioxetane phosphate was added to the membrane and incubated for 10 min at 37°C. All membranes were exposed for 10 min to an X-ray film.

### 5' RACE

Rapid amplification of 5' complementary DNA ends (5' RACE) was performed using the FirstChoice RLM-RACE kit (Ambion) according to the manufacturer's instructions, except that the CIP treatment at the start was omitted because prokaryotic RNA was used. A control without tobacco acid pyrophosphatase (TAP) treatment was included each time [13]. Sequences of gene specific inner and outer primers are listed in Additional file 4, Table S2. 40 PCR cycles were performed at 58°C using 2 μl reverse transcription reaction (SuperScript VILO cDNA Synthesis Kit, Invitrogen) and Taq polymerase (Westburg). 5' RACE products were analyzed using 2% agarose gels and the specific TAP treated products were cloned into pCRII-TOPO (Invitrogen). Between 5 and 10 clones carrying inserts of the expected size were sequenced.

## Results and Discussion

To systematically study the intergenic transcriptome of *R. etli *under diverse conditions, we opted to determine transcription profiles at various time points during free-living growth in defined medium as well as during the nitrogen-fixing endosymbiosis with its eukaryotic host plant *P. vulgaris*, totaling 5 sampling conditions. Identification of possible ncRNA elements was performed by comparing a comprehensive set of ncRNA predictions for various α-proteobacteria obtained from literature with our expression data (see Methods section). Table 1 gives an overview of the studies and the number of ncRNA predictions that were used as well as the number of detected ncRNAs sharing similarity with a predicted or verified ncRNA reported by each paper. Due to redundancy between the results of the respective studies, some of the reported candidate ncRNAs correspond to two or more predicted ncRNA (see Additional file 2, Table S1). Identified ncRNAs were classified based on whether they had been experimentally observed prior to this study, and if so, whether any functional characterization had been carried out (see Table 2). The results are summarized and discussed below, while details on individual expression levels and additional characteristics for each identified ncRNA are provided in Additional file 2, Table S1.

**Table 1:** Overview of the predicted and detected ncRNAs.

Reference	Organism	Predicted^(a)^	Detected^(b)^
del Val *et al*. 2007	*Sinorhizobium meliloti*	32(8)	11
Weinberg *et al*. 2007	α-proteobacteria	955	32
Livny *et al*. 2008	*Rhizobium etli*	189	60
Ulve *et al*. 2008	*S. meliloti*	67(14)	10
Valverde *et al*. 2008	*S. meliloti*	271(18)	29
Landt *et al*. 2008	*Caulobacter crescentus*	(300/27)	3

(a) Number of predicted ncRNAs that were used in this study. The number of ncRNAs detected in the respective study is indicated between brackets.

(b) Number of *R. etli *candidate ncRNAs detected in this study.

**Table 2:** Classification of the detected candidate ncRNAs^(a)^

	sRNA	*cis*-regulatory	Total
Functionally characterized	6	5	11
Uncharacterized	8	4	12
Novel candidate	17	49	66
	31	58	89

(a) See Additional file 2, Table S1 for a detailed summary.

### Functionally characterized ncRNAs

Expression of 4 sRNAs that are highly conserved among bacteria, including 6S RNA, the signal recognition particle RNA 4.5S (SRP), bacterial RNase P class A and tmRNA, was observed. Furthermore, several known riboswitches and replication incompatibility factors were also detected, thus providing a first validation of our approach to identify ncRNAs.

6S RNA is known to associate with RNA polymerase holoenzymes containing σ^70 ^[48,49], blocking σ^70^-dependent transcription during stationary phase when 6S is abundant. Transcription from many σ^70^-dependent promoters will be inhibited while transcription from σ^S^-promoters will increase. This is one mechanism that allows the stationary phase sigma factor σ^S ^(*rpoS*) to be an effective regulator in *E. coli *[6]. However, ε- and α-proteobacteria, including *R. etli*, do not have an *rpoS *homologue. Although an alternative mechanism functionally equivalent to *rpoS *is not known, 6S RNA is highly expressed in *R. etli *during the stationary phase and might therefore play a similar role as it does in *E. coli*.

SRP is a universally conserved ribonucleoprotein implicated in the translation and targeting of proteins to cell membranes. The SRP of most bacteria is composed of the Ffh protein and the 4.5S RNA molecule [50,51]. The expression of 4.5S RNA in *R. etli *is highest during stationary phase.

RNase P is an omnipresent endoribonuclease, found in bacteria, Archaea and Eukarya including mitochondria and chloroplasts. The processing of precursor-tRNAs into tRNAs with mature 5'-ends is its best characterized function [52]. RNase P of *R. etli *is highly expressed in both free-living and symbiotic conditions, but especially in the stationary phase.

tmRNA or SsrA directly affects gene expression in general. It rescues stalled ribosomes and tags incomplete polypeptides for degradation [53]. Even though the sensitivity to tmRNA defects varies with species and growth conditions, tmRNA seems to play a role in the ability of cells to adapt to and survive in diverse environments [6]. *R. etli *tmRNA is expressed in all conditions, but primarily in the stationary phase.

**Figure 2:**
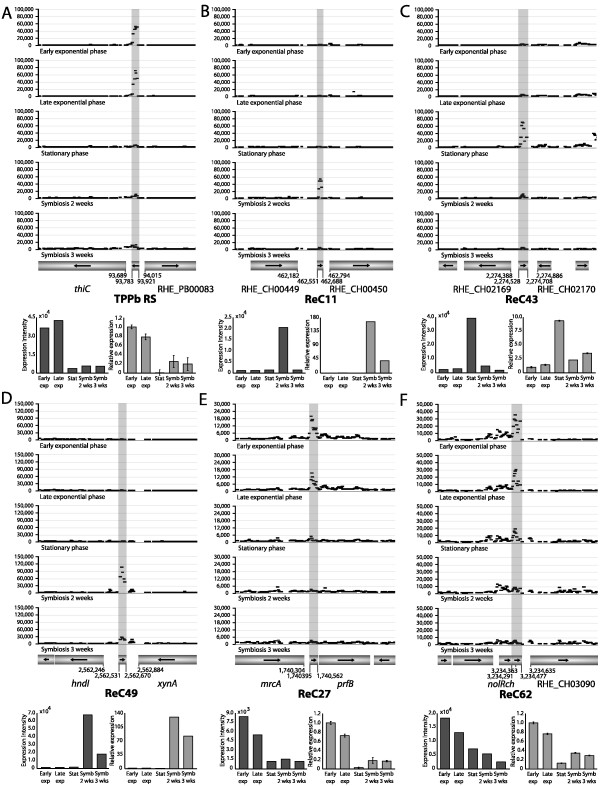
**Expression profiles of selected ncRNAs**. The individual probe intensities of six selected ncRNAs are shown during free-living growth (early exponential, late exponential and stationary phase) and symbiosis (two and three weeks after plant inoculation). The summary expression values of the probe intensities (left) and RT-qPCR confirmations of the ncRNA expression levels (right) are given below each profile. The mean values and standard deviation of three RT-qPCRs are shown. Genes are represented by bars and arrows indicate the direction of transcription. (A) TPPb RS is a thiamin pyrophosphate-sensing riboswitch located on plasmid p42B; (B) ReC11 is highly similar to a previously experimentally verified sRNA in *S. meliloti *1021 [41,45]; (C, D, E, F) ReC43, ReC49, ReC27, ReC62 are novel candidate ncRNAs.

Riboswitches (RS) are *cis*-regulatory RNAs located in the 5'-untranslated region (UTR) that directly sense the levels of specific metabolites [43]. Expression of five known RS was detected in *R. etli*: one glycine RS (containing two GCVT elements), one flavin mononucleotide RS (three RFN elements), one cobalamin RS (three B12 elements) and two thiamin pyrophosphate RS (two and three THI elements, respectively) [10,54]. One of the latter, TPPb RS (Figure 2A) has previously been shown to be indispensable for the regulation of the *thiCOGE *genes that are required for the de novo synthesis of thiamin in *R. etli *and other bacteria [55,56]. According to the proposed model, the *thiC *promotor is constitutive and the transcript is fully elongated when thiamin is absent. However, if sufficient thiamin is available, the transcript will be prematurely terminated at the putative attenuator located from +522 to +547. Therefore, it appears that under our conditions, thiamin is sufficiently present during exponential growth as the downstream operon is not transcribed. However, the length of the transcribed region does not fully support the model as a smaller region of 138 base pairs was detected. ReC76 is another TPP RS and shows the same expression pattern. The expression levels of the TPPb RS were confirmed by RT-qPCR. Finally, *incA *is a highly conserved small antisense RNA located between *repB *and *repC *and a strong incompatibility determinant of *repABC*-type plasmids [57,58]. All six plasmids of *R. etli *encode these *repABC *genes that control plasmid replication, segregation and copy number. The *incA *sequence is present in all plasmids, except for plasmid A. We detected expression of *incA *genes during stationary phase on the symbiotic plasmid and plasmid E. Why *incA *could only be detected on two of the five plasmids is unclear.

### Uncharacterized ncRNAs

12 ncRNAs are homologous to one or more previously identified but functionally uncharacterized ncRNAs in other α-proteobacteria. Remarkably, there is a sizeable overlap for this class of ncRNA between the results of the various predictive studies used. For example, ReC06 is similar to a prediction of five out of the six studies used, while ReC25, ReC58 and ReC59 were predicted by four out of six. This is partly due to three studies focusing on *Sinorhizobium meliloti *yielding redundant predictions using different computational approaches [41,42,45]. ReC11 (Figure 2B), for example, is similar to a *S. meliloti *sRNA highly expressed during symbiosis. This is also the case in *R. etli*, based on array data and as verified by RT-qPCR.

Three candidate ncRNAs share similarity with elements identified previously in *Agrobacterium tumefaciens *and other α-proteobacteria [59]. ReC58 and ReC59 are both similar to the regulatory RNA element *suhB*, an sRNA that is probably involved in antisense gene regulation. Neither ReC58 nor ReC59 is flanked by *suhB *homologues in *R. etli*. Also in accordance with previous detection in *A. tumefaciens*, ReC69 is located upstream of the *R. etli serCA*-operon.

### Novel ncRNAs

Our tiling array expression data provide experimental evidence for 66 ncRNAs that are homologous to previously predicted ncRNAs for which no experimental evidence was reported prior to this study. These candidate ncRNAs comprise 17 putative sRNAs and 49 putative *cis*-regulatory ncRNA elements. The majority of these novel ncRNAs, 48 in total, correspond to predictions of Livny *et al*. [44].

ReC43 and ReC49 are possible transcriptionally independent *trans*-regulatory sRNAs (Figure 2C, D). Both are highly expressed under specific conditions, respectively stationary phase and symbiosis. The expression levels of both sRNAs were confirmed by RT-qPCR. 7 of the 49 *cis*-regulatory ncRNA elements, including ReC27 (Figure 2E), have one or more regulatory elements that are known to depend on structured RNA, called riboswitch-like elements [46]. No expression was detected downstream of these putative RS, ReC33 and ReC41 excepted. This was also the case for the known RS (see above), suggesting that the RS-mediated regulation occurs mainly via transcription termination. This is somewhat unexpected as most described RS in Gram-negative bacteria function by inhibiting translation initiation [54].

The genes downstream of the remaining 42 novel *cis*-regulatory ncRNAs often showed a lower expression or no expression at all, indicating the presence of possible 5' UTR fragments giving rise to short transcripts. These sRNAs may have independent functions or, alternatively, be byproducts of (post-) transcriptional regulation. Similar findings were reported previously in *E. coli *[59-61] and *L. monocytogenes *[27]. ReC62 is an example of a ncRNA that overlaps the 3' UTR (Figure 2F).

A well-studied regulator in rhizobia is the RpoN sigma factor (σ^54^) that is required for nitrogen assimilation and nitrogen fixation during symbiosis in particular. *R. etli *contains two RpoN paralogs [62]. One is needed during free-living growth, the other during symbiosis. No significant matches to the RpoN binding site consensus sequence could be detected upstream of the detected candidate ncRNAs. Similarly, Livny *et al*. (2008) reported the absence of LexA, σ^54 ^and Fur binding sites upstream of predicted *R. etli *ncRNAs [44].

### Condition-specific expression and validation of array data

**Figure 3:**
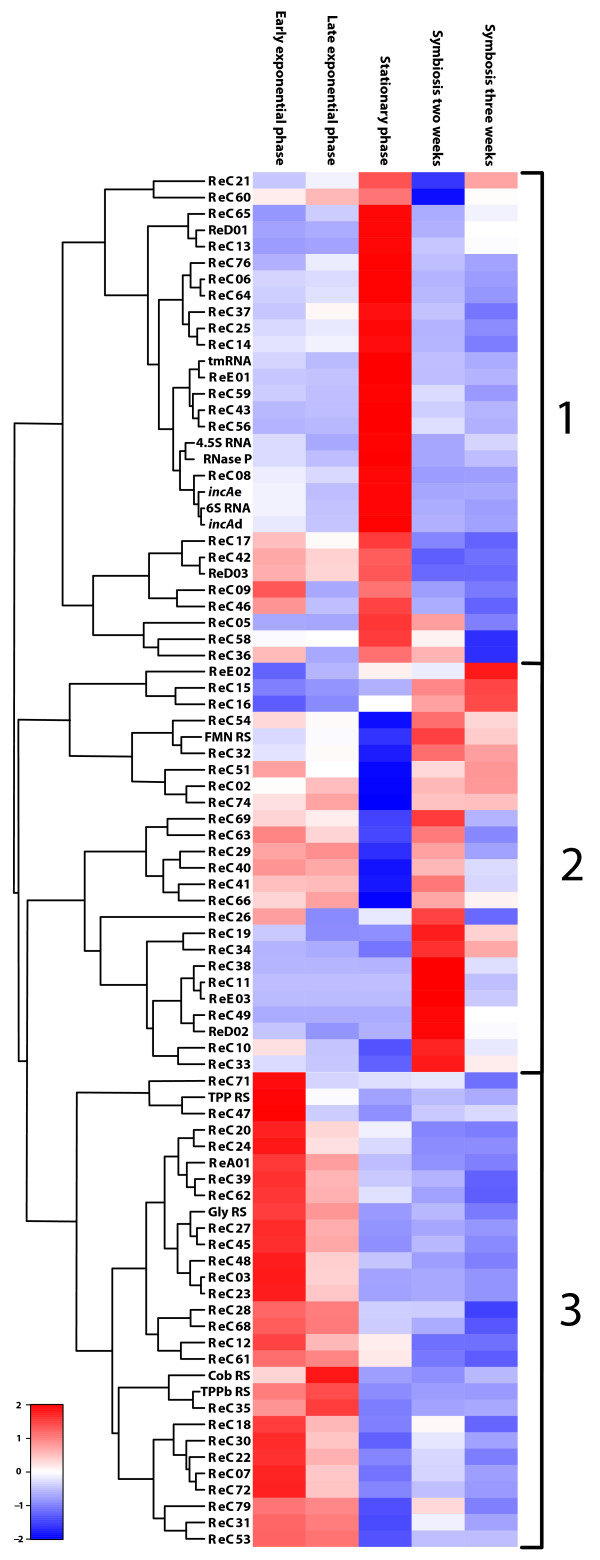
**Heat map of the candidate-ncRNAs**. The heat map visualizes the individual ncRNA transcription profiles of the detected ncRNAs that were differentially expressed under our experimental conditions. The expression values in each row were standardized by subtraction of the mean and division by the standard deviation and hierarchically clustered using R. ncRNAs showing similar expression patterns are grouped as follows: group 1, stationary phase; group 2, symbiosis; group 3, exponential phase. The letters b, d and e indicate gene location on the respective plasmids.

Numerous ncRNAs identified here are differentially expressed during free-living and symbiotic growth (Figure 3). By clustering these ncRNAs, 3 groups were identified containing candidate ncRNAs primarily expressed during exponential growth, stationary phase or symbiosis, respectively. Condition-specific gene expression often sheds light on a gene product's function. This was recently shown to hold true for ncRNAs as well. Toledo-Arana *et al. *identified a *L. monocytogenes *sRNA whose expression is specifically induced by blood serum. Importantly, a mutant strain unable to express the sRNA is severely attenuated in a mouse infection model [27]. We therefore anticipate that this clustering analysis yields prime targets for future functional characterization of ncRNAs with important roles in growth phase transition or symbiosis.

Differential expression of several ncRNAs (TPPb RS, ReC11, ReC27, ReC43, ReC49, ReC62) was independently confirmed using RT-qPCR, the data obtained using this complementary technique correlating well with the expression levels estimated from the array data (Figure 2).

**Figure 4:**
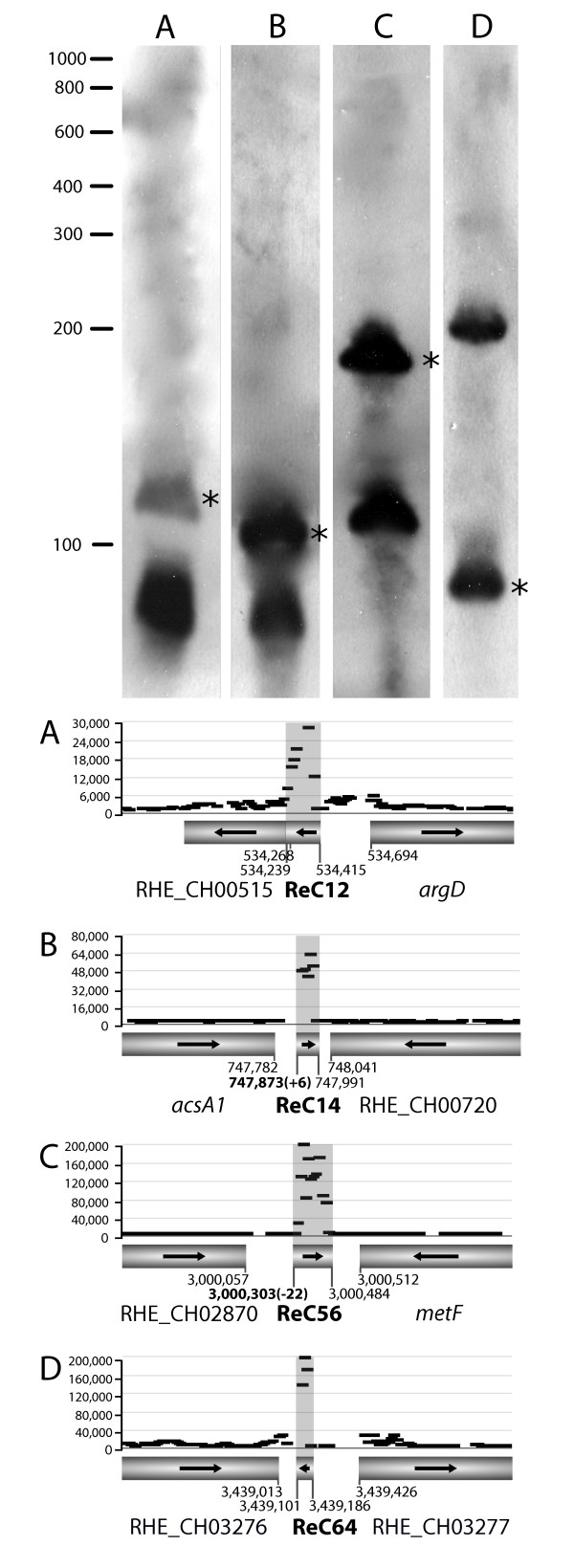
**Novel ncRNAs detected by Northern analysis**. Four novel ncRNAs were analyzed to validate the tiling array results. All four ncRNAs were detected during exponential and stationary phase grown in defined medium; expression during one growth phase is shown for each ncRNA. Primary transcripts are indicated by an asterisk. Transcription initiation sites identified by 5' RACE are shown in bold and the difference with the estimated transcription initiation sites using the array data are shown between brackets. Genes are represented by bars and arrows indicate the direction of transcription. (A) ReC12 and (B) ReC14 detected during early exponential phase; (C) ReC56 and (D) ReC64 detected during stationary phase.

To further validate our array data, the transcript lengths and transcription initiation sites of a selection of ncRNAs (ReC12, ReC14, ReC56, ReC64) were determined by Northern analysis and 5' RACE. The expected primary transcript lengths were readily observed by Northern blotting for ReC14, ReC56 and ReC64, illustrating the high resolution of the tiling array (Figure 4). The apparent size of ReC12 is slightly smaller than estimated. This overestimation is probably due to overlapping expression signals of the downstream gene. Further biochemical evidence was obtained by performing 5' RACE for ReC14 and ReC56. The experimentally determined transcription initiation sites are in good agreement with the array data (Figure 4).

In addition to the primary transcripts, a smaller band was detected for ReC12, ReC14 and ReC56 (Figure 4). These bands indicate endonucleolytic processing of the primary transcripts into smaller fragments. Processing is a common feature of ncRNAs. Stable sRNAs like 6S, 4.5S and tmRNA are observed to mature by 5'- and 3'-endonucleolytic cleavage, as is the case for other sRNAs such as RprA, SraC and SraG in *E. coli *[13]. For ReC64, an additional transcript, larger than expected was observed. It is unclear where this fragment might have originated from and why it was not detected on the array. Evidence for processing was also observed during 5' RACE experiments, as a less specific signal was detected in the RNA samples that were not treated with TAP. Treatment with and without TAP allows the distinction between 5'-triphosphate ends (transcriptional initiation sites) and 5'-monophosphates generated by processing of primary transcripts. Therefore, the processing likely occurs upstream of the inner RACE primer. The processed RACE products were not analyzed in this study. A more detailed analysis in the future could clarify the precise processing sites.

### Genomic distribution and conservation in other α-proteobacteria

**Figure 5:**
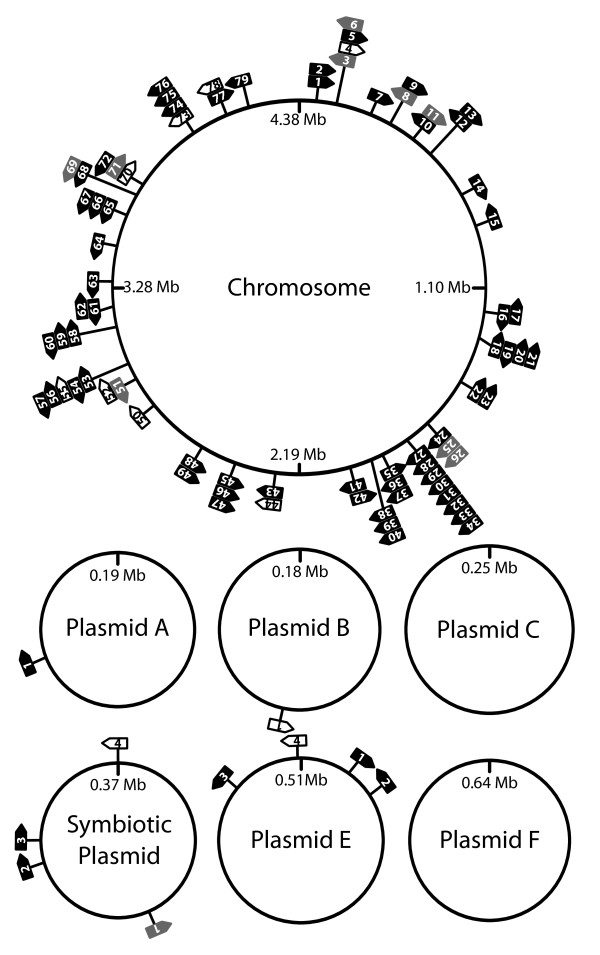
**Genomic map of ncRNAs**. White, grey and black arrows show the location of functionally characterized, previously reported but uncharacterized and novel ncRNAs, respectively. The majority of the identified ncRNAs are chromosomally encoded. An apparent 'hot spot' with increased ncRNA gene density is located around 1.7 to 2.0 Mb on the chromosome.

The identified candidate ncRNAs are located on the chromosome as well as on 3 of the 6 megaplasmids comprising the 6.5 Mbp *R. etli *genome (Figure 5). Nearly 90% of the ncRNAs can be found on the chromosome, averaging 18 ncRNAs per megabase compared to less than 5 ncRNA elements per megabase of plasmid DNA. It is unclear at present whether this observation is a consequence of the computational approaches used to predict the ncRNAs, of the relatively limited number of conditions used to evaluate ncRNA expression, or whether there is indeed a bias in genomic ncRNA location. In support of the latter hypothesis, a similar observation was reported for *S. meliloti *[45]. As can be seen from the graphical representation of the genomic ncRNA distribution, an apparent ncRNA 'hot spot' enriched for detected ncRNA elements is situated around 1.7 to 2.0 Mb on the chromosome (Figure 5).

**Figure 6:**
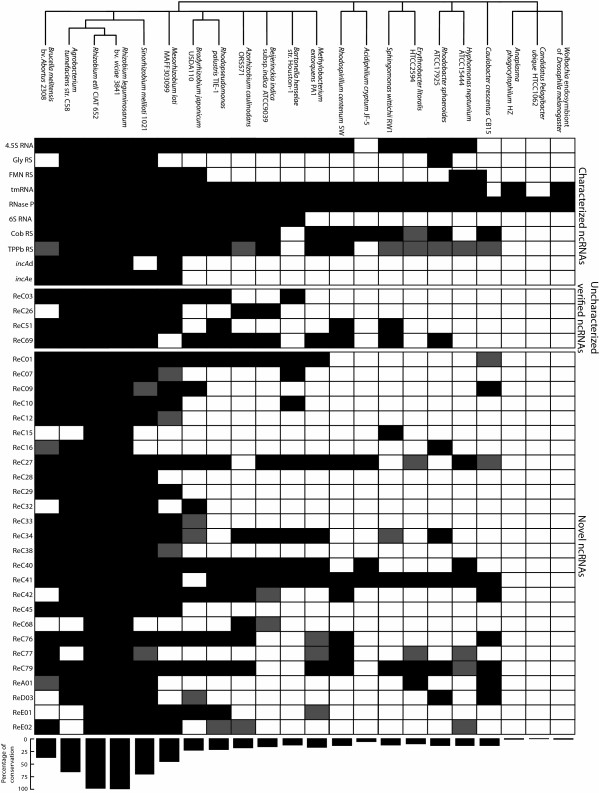
**Conservation of *R. etli *ncRNAs in α-proteobacteria**. Similarity analysis was performed using BLASTN (NCBI). Black squares indicate E-values ≤ 10^-5^, grey squares represent E-values ≤ 10^-3^. The percentage of conservation for each species is shown at the bottom of the figure. Only ncRNAs conserved beyond *A. tumefaciens *str. C58, *R. etli *CIAT652, *R. leguminosarum *bv. *viciae *3841, *S. meliloti *1021 and *Mesorhizobium loti *MAFF303099 are shown. Conservation analysis results of all identified ncRNAs are included as supplementary data, see Additional file 5, Table S3.

In order to examine the conservation of the detected ncRNAs, we performed a BLASTN search of all candidate ncRNAs against the genome sequence of at least one member of each family of the α-proteobacteria. While primary sequence similarity of the different ncRNAs is mostly limited to closely related species such as *A. tumefaciens*, *Rhizobium leguminosarum*, *S. meliloti *and *R. etli *CIAT652, 40 out of the 89 ncRNAs were found to be conserved in more distantly related species as well (Figure 6 and Additional file 5, Table S3). These results should be interpreted with care, however, as ncRNA is notoriously variable at the primary sequence level [24]. The lack of sequence similarity between functional ncRNA homologues was also observed in other bacteria. The *E. coli *sRNAs CsrB and CsrC show little homology with their counterparts in *Vibrio fischeri*, and *P. aeruginosa *RhyB shows minimal similarity with its *E. coli *homologue [63,64]. Therefore, the conservation reported here could be a severe underestimation of the actual figure.

## Conclusions

In recent years, a number of studies were published predicting ncRNAs in many α-proteobacteria including *R. etli*. We therefore decided to put these predictions to the test by combining *R. etli *tiling array expression data and a comprehensive analysis of a large number of predicted ncRNAs. This allowed us to detect 89 ncRNAs out of 447 candidate ncRNA regions. Undoubtedly, there are still many more ncRNAs to be discovered. Improvements in computational analyses and the inclusion of more experimental conditions will surely contribute to this number, as will de novo ncRNA discovery starting from the expression data.

We were able to discern well-characterized ncRNAs like 6S RNA, tmRNA and a TPP RS, several previously reported but uncharacterized ncRNAs, as well as a large number of novel ncRNAs similar to earlier predictions that had not been detected experimentally before. Additional experimental evidence for the detected ncRNAs was obtained by Northern analysis for 4 novel ncRNAs and two of them were also verified by 5' RACE. Our results show an endonucleolytic processing of the selected ncRNAs. Conservation analysis showed that a significant number of ncRNAs is conserved beyond closely related species. With condition-specific expression patterns providing a first clue to the role that some of these ncRNAs may play, a further functional analysis will help to better understand the intricate details of ncRNA-mediated gene regulation allowing bacteria to adapt to different and alternating environmental conditions.

## Authors' contributions

MV performed the experiments and bioinformatics analysis. MV, MF and JM conceived the study and contributed to the interpretation of the data. LC, KE, IMT and KM performed and contributed to the microarray normalization. MV, MF and JM were involved in drafting the manuscript. All authors read and approved the final manuscript.

## Supplementary Material

Additional file 1 **Figure S1**. The probability density functions of the microarray data for each condition, used to determine the expression significance threshold. Click here for file

Additional file 2 **Table S1**. List of all 89 candidate ncRNAs with detailed additional information, including quantitative expression data. Click here for file

Additional file 3 **Figure S2**. The probability density functions over all five conditions, used to determine differentially expressed ncRNAs. Click here for file

Additional file 4 **Table S2**. List of Northern blot probes and 5' RACE primers. Click here for file

Additional file 5 **Table S3**. Conservation analysis of all identified ncRNAs based on similarity analysis within the α-proteobacteria. Click here for file
