# Supplementary figures and images for: Genome-wide detection of predicted non-coding RNAs in Rhizobium etli expressed during free-living and host-associated growth using a high-resolution tiling array
Source: BMC Genomics. 2010 Jan 20;11:53. doi: 10.1186/1471-2164-11-53 (PMC2881028; doi:10.1186/1471-2164-11-53)

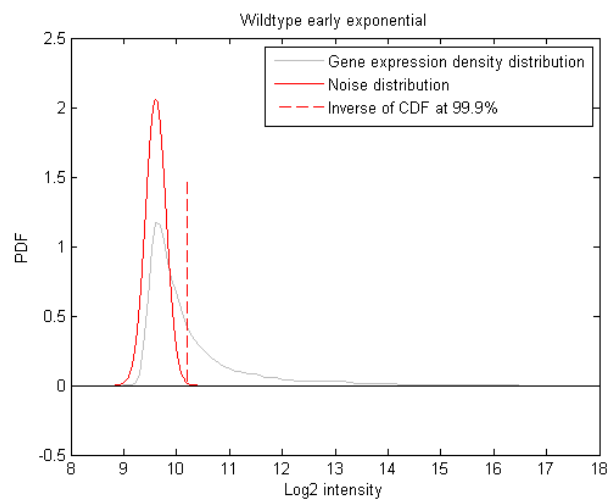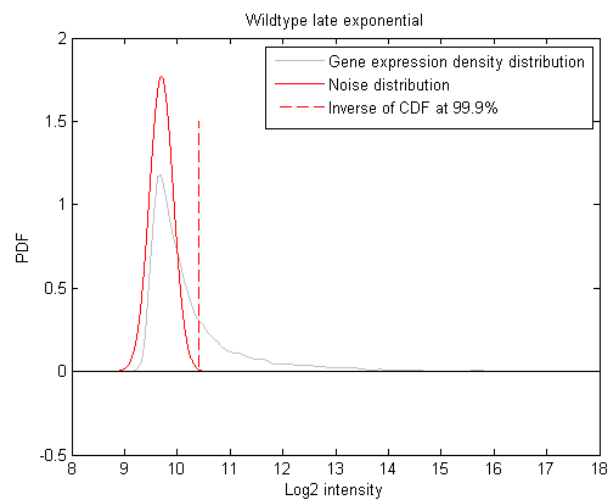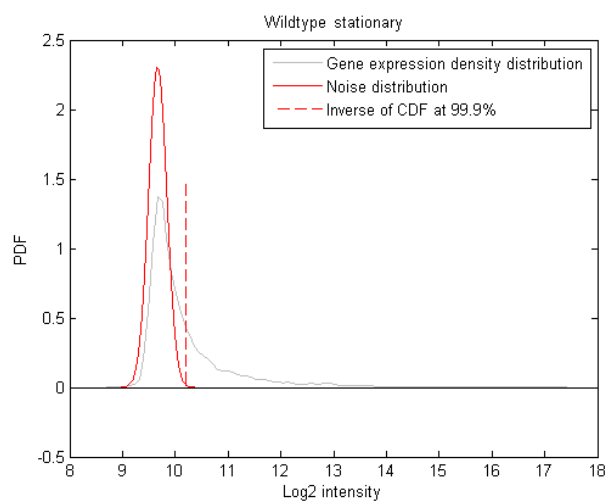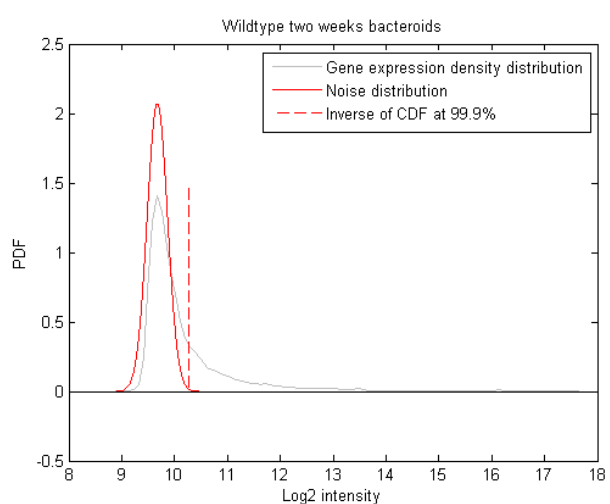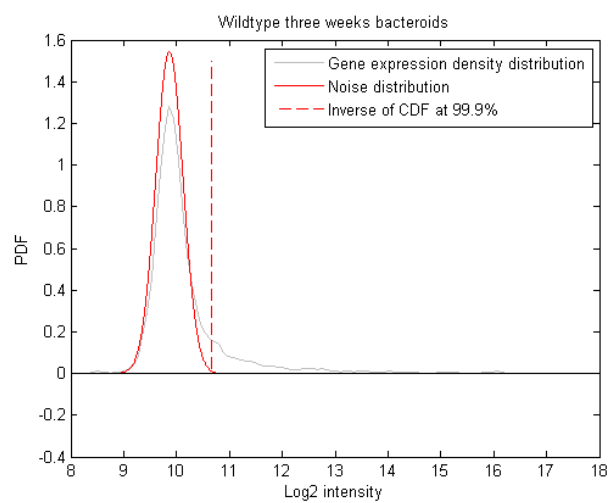

Supplement: Additional file 1 — Figure S1. The probability density functions of the microarray data for each condition, used to determine the expression significance threshold. [file 1471-2164-11-53-S1.pdf]

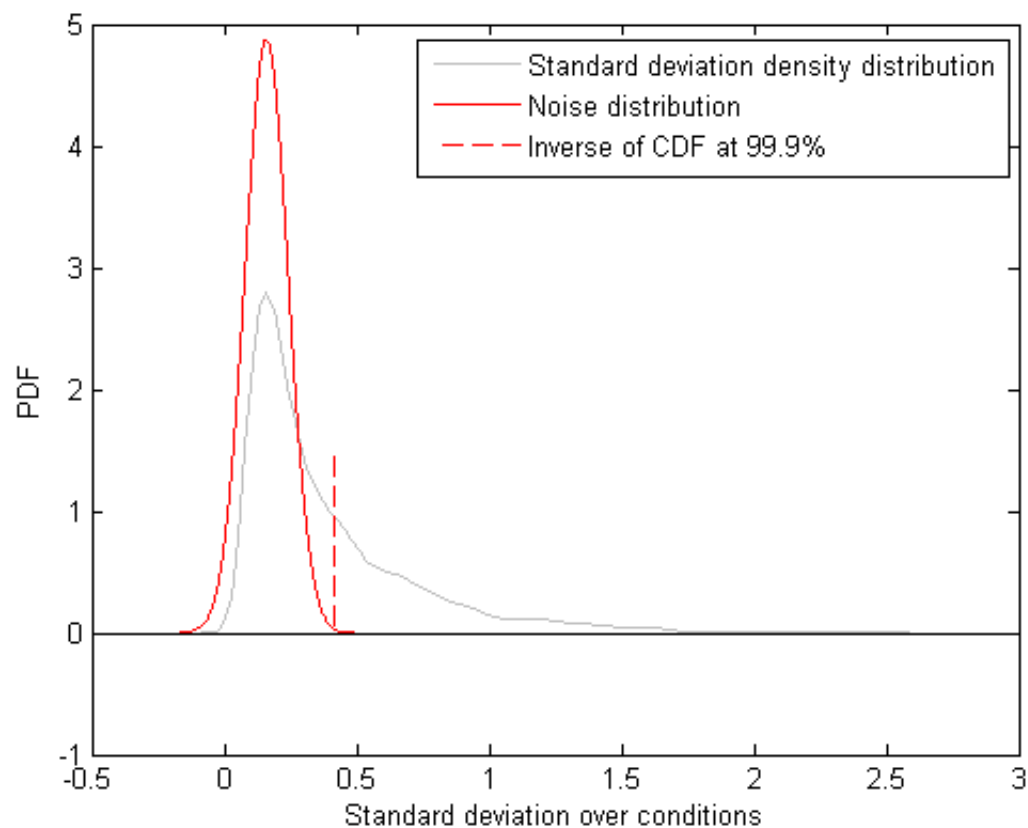

Supplement: Additional file 3 — Figure S2. The probability density functions over all five conditions, used to determine differentially expressed ncRNAs. [file 1471-2164-11-53-S3.pdf]
